# Supplementary material for: Oxidative Stress Modulation by Cameroonian Spice Extracts in HepG2 Cells: Involvement of Nrf2 and Improvement of Glucose Uptake
Source: Metabolites. 2020 May 1;10(5):182. doi: 10.3390/metabo10050182 (PMC7281205; doi:10.3390/metabo10050182)
Supplement: Supplementary file 1 [file metabolites-10-00182-s001.zip › Figure 1S.pptx]

## Slide 1
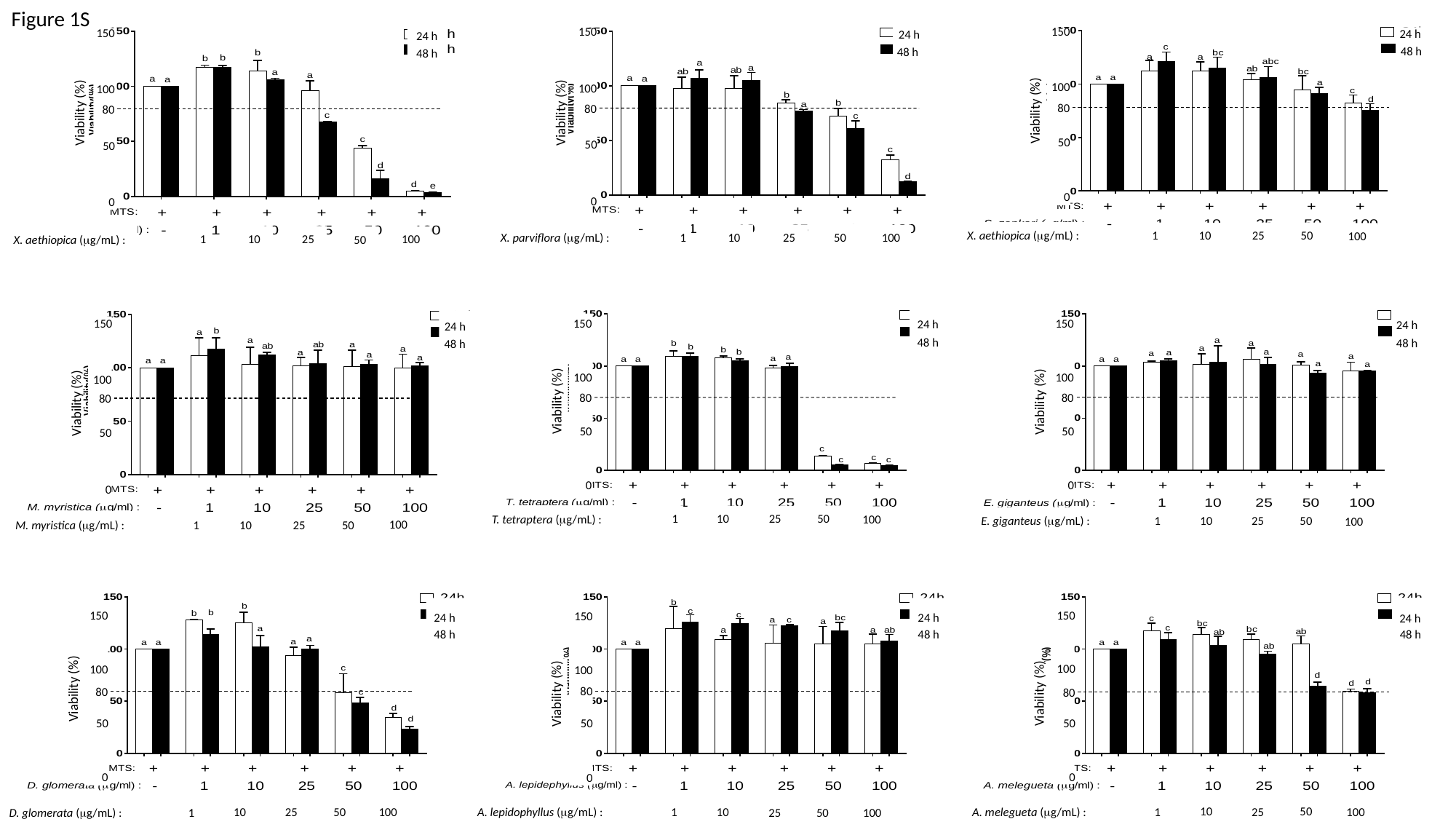

Figure 1S
150
150
150
24 h
24 h
24 h
48 h
48 h
48 h
100
100
100
Viability (%)
80
Viability (%)
80
Viability (%)
80
50
50
50
0
0
0
X. aethiopica (mg/mL) :
25
50
1
10
100
X. parviflora (mg/mL) :
50
10
25
1
100
X. aethiopica (mg/mL) :
10
25
1
100
50
150
150
150
24 h
24 h
24 h
48 h
48 h
48 h
100
100
100
Viability (%)
Viability (%)
80
80
80
Viability (%)
50
50
50
0
0
0
T. tetraptera (mg/mL) :
50
25
10
1
100
E. giganteus (mg/mL) :
25
50
10
1
100
M. myristica (mg/mL) :
100
50
10
25
1
150
150
150
24 h
24 h
24 h
48 h
48 h
48 h
100
100
100
Viability (%)
Viability (%)
Viability (%)
80
80
80
50
50
50
0
0
0
A. melegueta (mg/mL) :
50
A. lepidophyllus (mg/mL) :
10
25
1
50
25
100
10
10
1
100
100
25
50
1
D. glomerata (mg/mL) :

## Slide 2
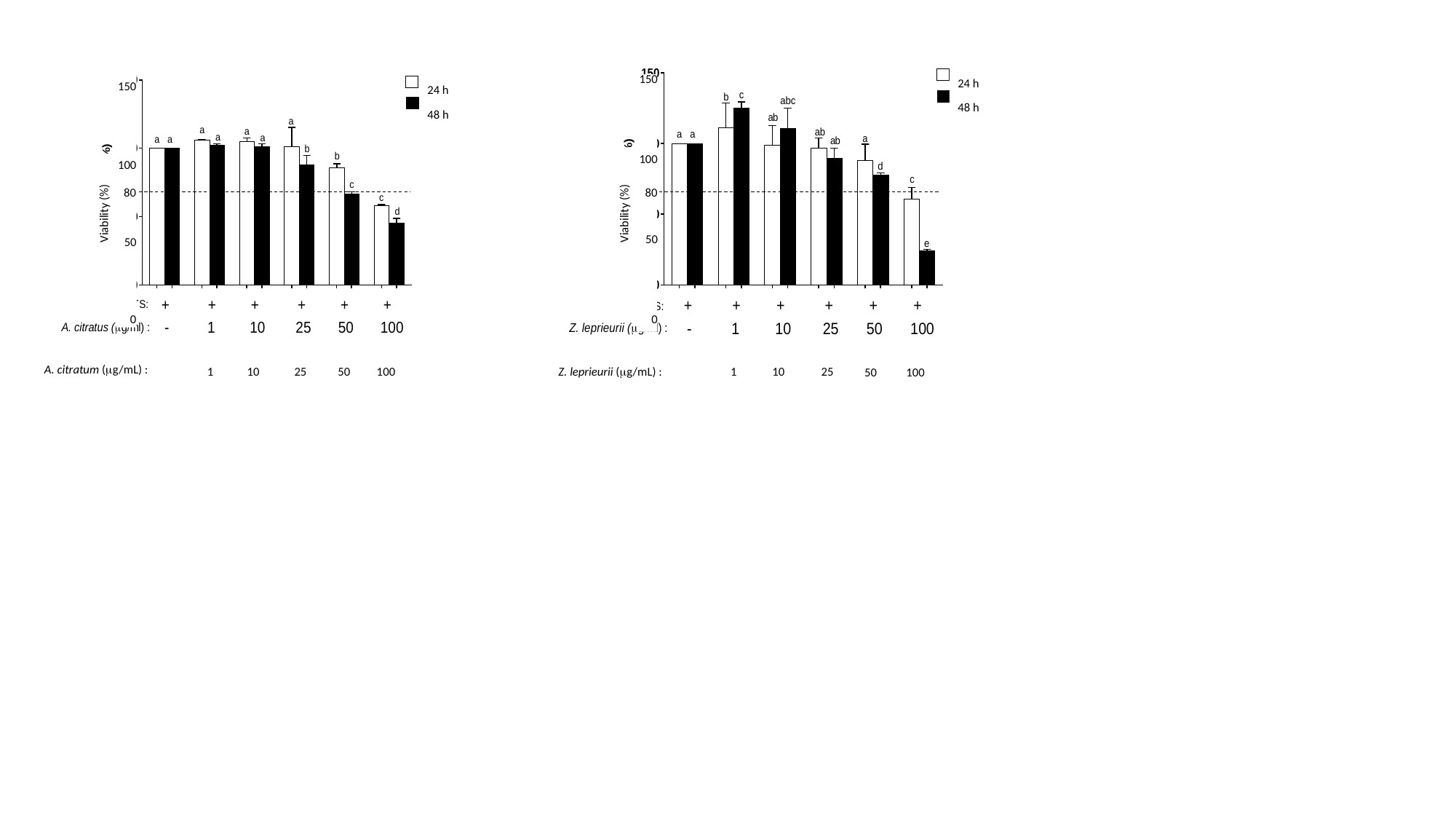

150
24 h
150
24 h
48 h
48 h
100
100
80
80
Viability (%)
Viability (%)
50
50
0
0
A. citratum (mg/mL) :
Z. leprieurii (mg/mL) :
10
25
1
100
50
10
25
1
100
50
