# Supplementary figures and images for: Oxidative Stress Modulation by Cameroonian Spice Extracts in HepG2 Cells: Involvement of Nrf2 and Improvement of Glucose Uptake
Source: Metabolites. 2020 May 1;10(5):182. doi: 10.3390/metabo10050182 (PMC7281205; doi:10.3390/metabo10050182)

## Slide 1
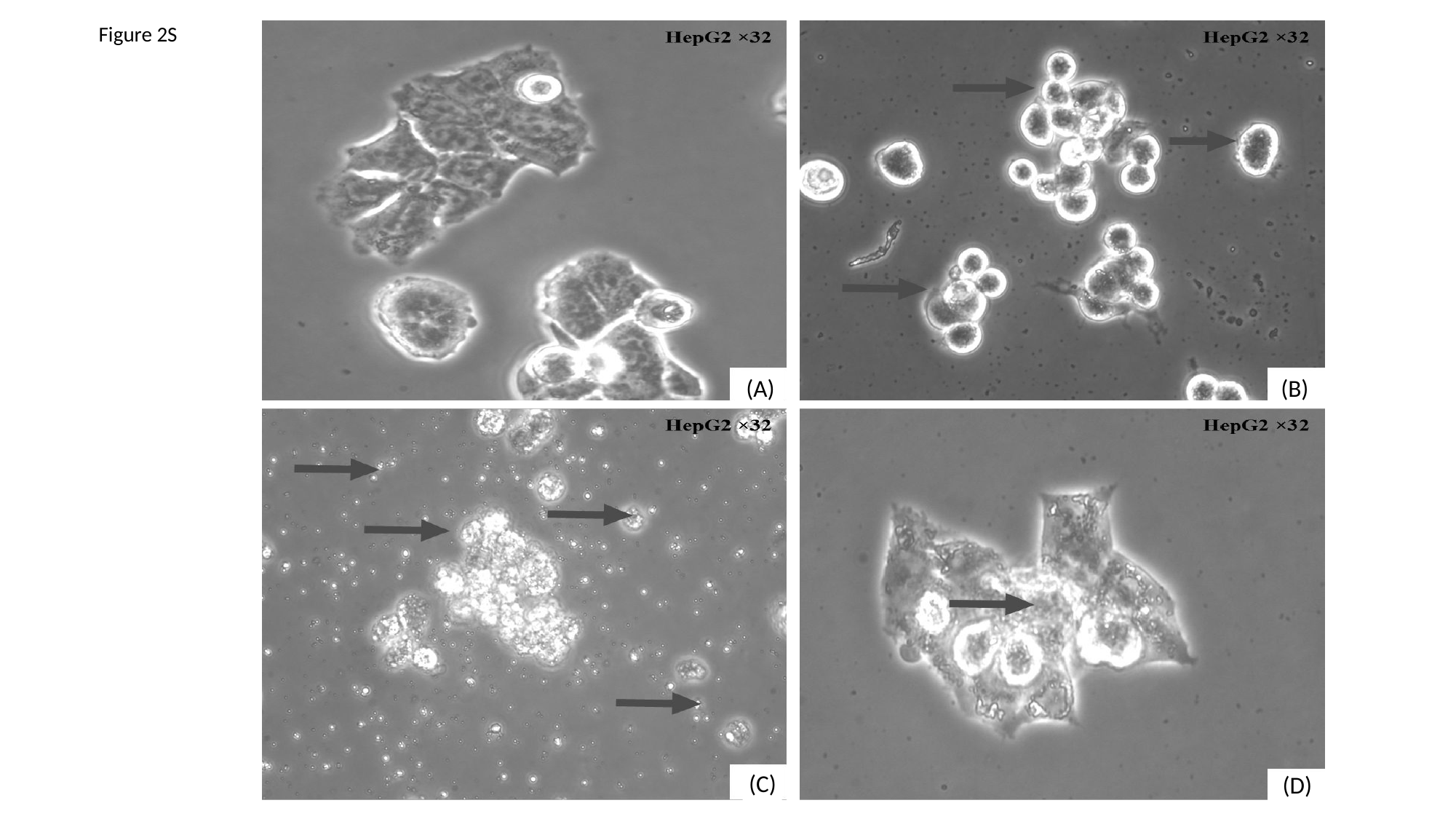

Figure 2S
(A)
(B)
(C)
(D)

Supplement: Supplementary file 1 [file metabolites-10-00182-s001.zip › Figure 2S.pptx]
